# Evaluation of the Accuracy of Current Tubeless Pumps for Continuous Subcutaneous Insulin Infusion

**DOI:** 10.1089/dia.2020.0525

**Published:** 2021-04-20

**Authors:** Ralph Ziegler, Nick Oliver, Delia Waldenmaier, Jochen Mende, Cornelia Haug, Guido Freckmann

**Affiliations:** ^1^ Diabetes Clinic for Children and Adolescents, Muenster, Germany.; ^2^ Division of Diabetes, Endocrinology and Metabolism, Imperial College London, London, United Kingdom.; ^3^ Institut für Diabetes-Technologie, Forschungs- und Entwicklungsgesellschaft mbH an der Universität Ulm, Ulm, Germany.

**Keywords:** Continuous subcutaneous insulin infusion, Insulin pump, Tubeless pump, Patch pump, Accuracy, Occlusion

## Abstract

***Background:*** Recently two new tubeless pumps for insulin therapy were introduced. They were tested for accuracy and occlusion detection and compared with the established patch pump Omnipod^®^ (OP).

***Methods:*** Using a modified setup for tubeless pumps based on IEC 60601-2-24, the basal rate and bolus delivery of the Accu-Chek^®^ Solo micropump system (ACS) and the A6 TouchCare^®^ System (A6) were measured with a microgravimetric method. Bolus sizes of 0.2, 1, and 10 U, and basal rates of 0.1 and 1 U/h were evaluated in nine repetitions. For each parameter, mean deviation and number of individual boluses or 1-h basal rate windows within ±15% from target were calculated. In addition, occlusion detection time at basal rates of 0.1 and 1 U/h was determined.

***Results:*** Mean deviation of boluses of different volumes in the pumps ranged from −3.3% to +4.0% and 40%–100% of individual boluses were within ±15% of the target. During basal rate delivery, 48% to 98% of 1-h windows were within ±15% of the target with a mean deviation between -5.3% and +6.5%. In general, considerable differences between pump models were observed and deviations decreased with increasing doses. In most parameters, ACS was more accurate, and A6 less accurate, than OP. Mean occlusion detection time ranged from ∼3 to 7.5 h at 1 U/h and was >24 h or absent at 0.1 U/h.

***Conclusions:*** In this evaluation, significant differences between the tested tubeless pump models were observed that became most evident when regarding delivery errors over short time and small volumes.

## Introduction

Continuous subcutaneous insulin infusion (CSII) is an effective method of insulin therapy in people with type 1 diabetes.^1^ Especially in children with diabetes, CSII is the method of choice.^2^ It allows a continuous basal insulin flow and additional bolus administration, whereby small frequent insulin microdoses can be administered and flexibly adjusted. Insulin pumps are also used in closed loop systems that automatically adapt insulin delivery to current glucose levels.^3^

Although evidence regarding clinical relevance is scarce, accuracy and reliability of insulin delivery by insulin pumps are considered important and have been tested by different study groups in recent times.^4–11^ In this context, there have been comparisons of tubeless pumps, also known as patch pumps, and durable pumps with one tubeless pump often showing inferior accuracy.^5,7,10,11^ However, the selection of tubeless pumps is more limited compared with durable pumps, leading to the first available tubeless pump being most frequently used as a comparator. This pump, though, might not be representative for current generation tubeless pumps. Although most durable pumps work according to the same principle, different mechanisms are utilized in tubeless pumps.^12^ The established patch pump Omnipod^®^ (OP) (Insulet) comprises a remote control and a disposable pod that contains the engine, the reservoir, and the cannula. It is driven by a shape memory alloy wire technology.^13^ The new A6 TouchCare^®^ System (A6) (Medtrum Technologies, Inc., Shanghai, China) has a reusable pump base containing electronics and a disposable reservoir including the cannula in addition to a remote control. The Accu-Chek^®^ Solo micropump system (ACS) (Roche Diabetes Care GmbH, Mannheim, Germany) is composed of a remote control, a reusable pump base with a piston-driven step motor, a disposable reservoir, and a disposable pump holder including the cannula.^14^

Accuracy testing of tubeless pumps is challenging, as the relevant standard IEC 60601-2-24 does not stipulate a suitable setting when no infusion set is available.^15^ Methods were previously established that are suitable for testing the delivery accuracy of tubeless pumps, and for comparing different types of pumps.^16^ However, to date, different test setups might have contributed to different levels of accuracy reported for tubeless and durable pumps. The present evaluation focuses exclusively on tubeless pumps, assessed with previously established methods.

## Methods

**FIG. 1.**
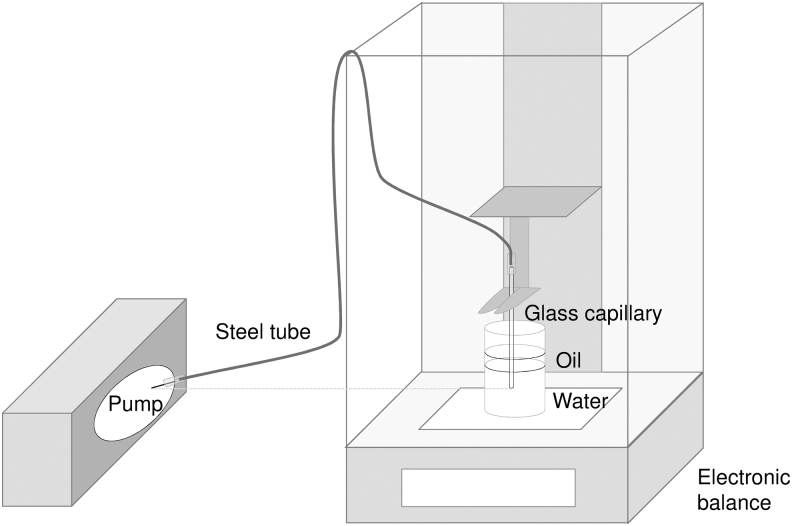
Setup for tests with tubeless pumps.^16^

In this investigation, two new tubeless insulin pumps, the ACS (Roche Diabetes Care GmbH) and the A6 (Medtrum Technologies, Inc.) were tested. All tests were performed in vitro, no human subjects were recruited and, therefore, no approval from an institutional review board was required. Bolus and basal rate delivery were measured in a microgravimetric system based on IEC 60601-2-24^15^ using a setup with a steel tube to connect the tubeless pump to the measuring vessel (Fig. 1). This setup has been tested with another tubeless pump and was shown to be suitable.^16^ For bolus accuracy testing, 25 successive 0.2 or 1 U boluses or 12 successive 10 U boluses of insulin aspart (NovoRapid^®^, Novo Nordisk A/S, Bagsværd, Denmark) were applied and weighed separately. In addition, the time for the complete delivery of a 10 U bolus was measured. Each set of measurements was repeated with three pumps (reusable parts and remote controls) and three disposables each, resulting in nine measurement sets available for evaluation. Constant basal rates of 0.1 and 1 U/h were delivered for 72 h and also repeated with 3 × 3 measurement sets. For the evaluation, basal rate runtime was divided into 1-h windows.

For both bolus and basal rate accuracy, mean total deviation from the target delivery including standard deviation (SD) and the percentage of individual boluses or 1-h basal rate windows that fell within ±15% from the target were calculated.

In a separate experiment, occlusion detection time during basal rates of 0.1 and 1 U/h was measured as described before.^17^ The soft cannula of ACS was occluded using a metal clamp and the steel cannula of A6 was occluded with a rubber cap. Occlusion alarm tests were limited to a maximum observation time of 72 h to ensure comparability with previous experiments^17^ and repeated nine times per pump.

The established tubeless pump OP (Eros version, Insulet Corporation, Acton, MA) was previously tested^7,10^ and these data were used for comparative analysis. However, as the minimum common bolus size for all three pumps, which is 0.2 U, was not tested before, this bolus size was tested with OP in the course of the tests with the new pumps. Although so far OP was only compared with durable pumps, the current analyses included a comparison of the three tubeless pumps based on previous and recent tests with a consistent validated methodology.

The comparative analysis of all three pumps included *χ*^2^-tests on the frequency of values (individual boluses and 1-h basal rate windows, respectively) within ±15% of the target. Adjusting for multiple testing, *P*-values <0.025 were regarded as statistically significant. In addition, mean absolute relative differences (MARD) between actual and target delivery were calculated for bolus and basal rate accuracy of each pump. Although MARD is usually used as an accuracy parameter for continuous glucose monitoring systems, it was recently introduced for insulin pumps.^11^ This parameter combines systematic and random errors and can, therefore, be implemented to compare different systems using a single reported value.

## Results

### Bolus accuracy

Accuracy of bolus delivery was tested for bolus volumes of 0.2, 1, and 10 U and is described by the mean deviation from target of all boluses, as well as the percentage of individual boluses within the range of ±15% of the target (Table 1). The mean deviation varied depending on the bolus volume and ranged from −3.3% to +4.0%. SDs as a measure for deviations between individual boluses showed considerable differences, especially for the 0.2 U bolus deliveries for which SD ranged from 8.4% (ACS) to 23.3% (A6). Supplementary Table S1 shows results for each of the nine measurements. Some variation between the used disposables was observed. In general, relative deviations were lower with the larger boluses, resulting in more values within ±15% of the target. Testing the 10 U bolus, all boluses from all pumps were within ±15% of the target, whereas only 40% (for A6) to 88% (for ACS) of 0.2 U boluses were within this range. Almost all 1 U boluses were within ±15% of target for ACS, whereas this applied only to 65% of boluses for A6. The mean time for a 10 U bolus to be completely delivered was 4:00 min for ACS and 6:38 min for A6.

**Table 1.** Accuracy of Bolus Delivery at Different Bolus Volumes (*n* = 225 for 0.2 and 1 U; *n* = 118 for 10 U)

Insulin pump	0.2 U		1 U		10 U	
	Mean deviation ± SD	Individual boluses within ±15% of the target	Mean deviation ± SD	Individual boluses within ±15% of the target	Mean deviation ± SD	Individual boluses within ±15% of the target
ACS	−3.3% ± 8.4%	88%	+0.3% ± 5.5%	99%	±0.0% ± 0.8%	100%
A6	+3.3% ± 23.3%	40%	+4.0% ± 16.0%	65%	+3.1% ± 0.8%	100%
OP	+1.5% ± 20.8%	57%	±0.0% ± 12.5%^7^	77%^7^	+0.3% ± 0.7%^7^	100%^7^

A6, A6 TouchCare^®^ System; ACS, Accu-Chek^®^ Solo micropump system; OP, Omnipod^®^; SD, standard deviation.

### Basal rate accuracy

**FIG. 2.**
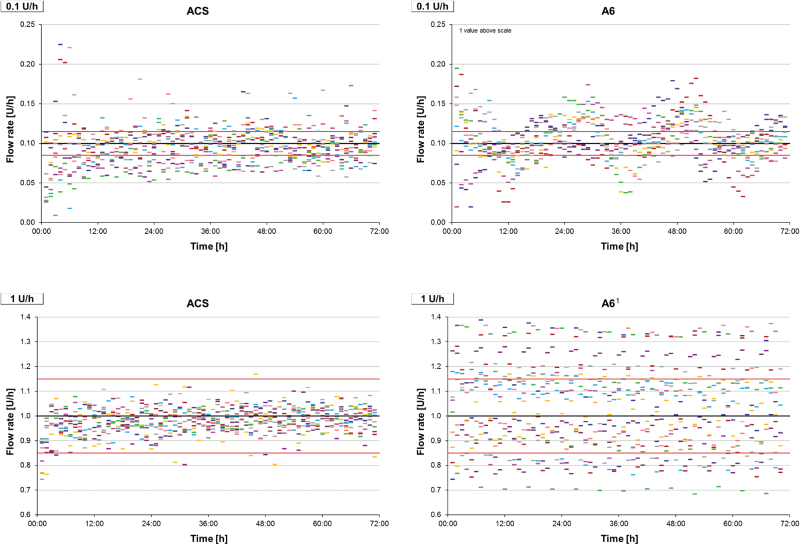
Accuracy of 1-h windows during 72 h basal rate delivery of 0.1 U/h (above) and 1 U/h (below). The black line represents target delivery, red lines represent target delivery ±15%, colored dashes represent the 1-h windows of individual measurements (nine repetitions). ^1^One measurement was stopped earlier due to an error notice.

Basal rate delivery was evaluated for 0.1 and 1 U/h for 72 h. Accuracy was higher when using a larger rate, in particular regarding the deviation between the individual 1-h windows (Table 2). Using the 0.1 U/h basal rate, approximately half of the 1-h windows were found within the range of ±15% of the target and both pumps showed similar SDs of >20%. Regarding the whole observation period, ACS showed a negative bias on average, whereas A6 showed a positive bias. Considering the course of delivery over time, ACS showed a constant delivery after a short run-in phase, especially at the 1 U/h basal rate (Fig. 2). A6, however, showed larger deviations between successive 1-h windows with apparent periodic variability. Results for individual measurements are shown in Supplementary Table S2. Differences between the individual devices became obvious when testing the small basal rate.

**Table 2.** Accuracy of Basal Rate Delivery at Different Basal Rates of Nine Measurements for 72 h Divided Into 1-h Windows

Insulin pump	0.1 U/h			1 U/h		
	Mean total deviation	SD (1 h windows)	1-h windows within ±15% of the target	Mean total deviation	SD (1 h windows)	1-h windows within ±15% of the target
ACS	−5.3%	25.0%	51%	−1.9%	5.4%	98%
A6	+4.8%	28.4%	48%	+3.4%^a^	17.0%	60%

^a^ One measurement was stopped earlier due to an error notice.

### Occlusion detection time

The time between manual occlusion of the cannula tip and the occurrence of an occlusion alarm was measured during basal rates of 0.1 and 1 U/h. The mean occlusion detection time was ∼3 h for ACS and 7.5 h for A6 when a 1 U/h basal rate was active (Table 3). With the smaller basal rate, ACS gave an alarm after a mean of ∼35 h and for A6 no alarm occurred within 72 h in any of the nine measurements.

**Table 3.** Occlusion Detection Time at Different Basal Rates (Mean of Nine Measurements)

Insulin pump	0.1 U/h (hh:mm)	1 U/h (hh:mm)
ACS	35:11	02:57
A6	9 × no alarm	07:26

### Comparative analysis

For a comparative analysis of current tubeless pumps, previously published data for a third and established tubeless pump were included along with a new evaluation of bolus and basal rate delivery, as well as occlusion detection time.^7,10,17^

**FIG. 3.**
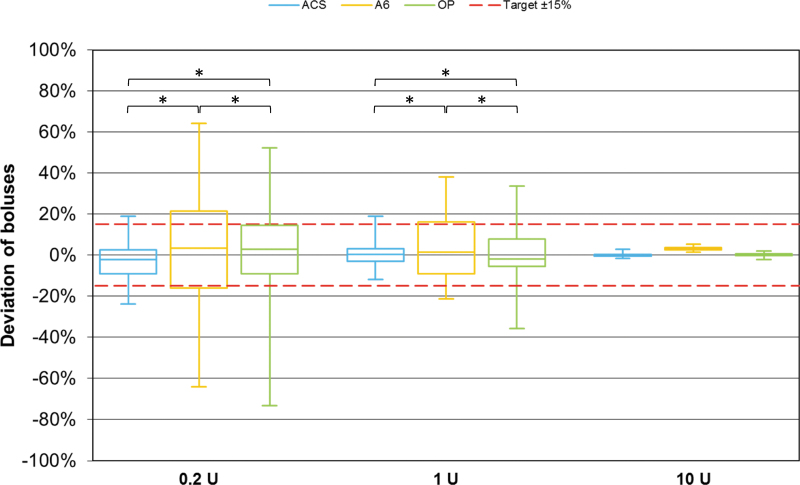
Bolus delivery accuracy of different pumps (*n* = 225 boluses for 0.2 and 1 U, *n* = 108 boluses for 10 U from nine repetitions per pump) grouped by bolus size. Red dashed lines represent target delivery ±15%. Asterisks indicate statistically significant differences in percentage of individual boluses within ±15% of the target between pumps (*χ*^2^-tests; *P* < 0.025).

The frequency of bolus values within a range of ±15% of the target was significantly greater with ACS than with A6 and OP for 0.2 and 1 U boluses (*P* < 0.001), and OP was more accurate than A6 (0.2 U: *P* < 0.001; 1 U: *P* = 0.007) (Fig. 3). For 10 U bolus deliveries, there was no significant difference.

**FIG. 4.**
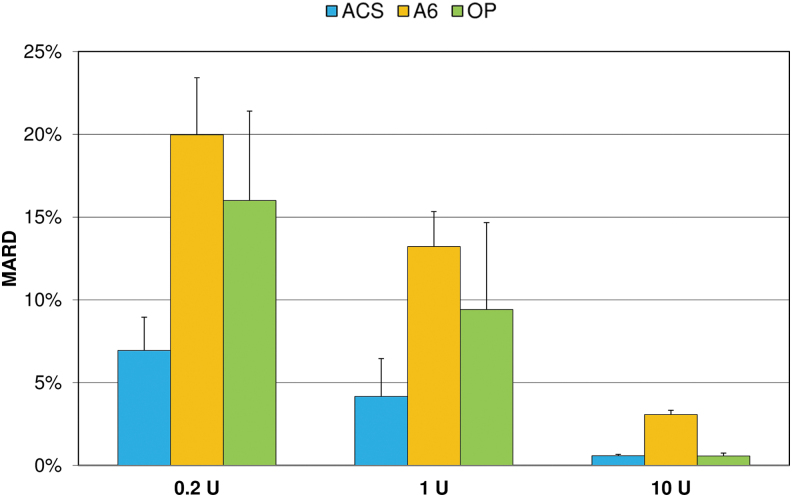
Mean absolute relative differences (MARD) of boluses from target delivery (mean and SD of nine repetitions). SD, standard deviation.

MARD values ranged from 0.6% to 20.0% and decreased with increasing bolus volumes in all pumps (Fig. 4). For all bolus volumes, MARD values were lowest with ACS and highest with A6.

**FIG. 5.**
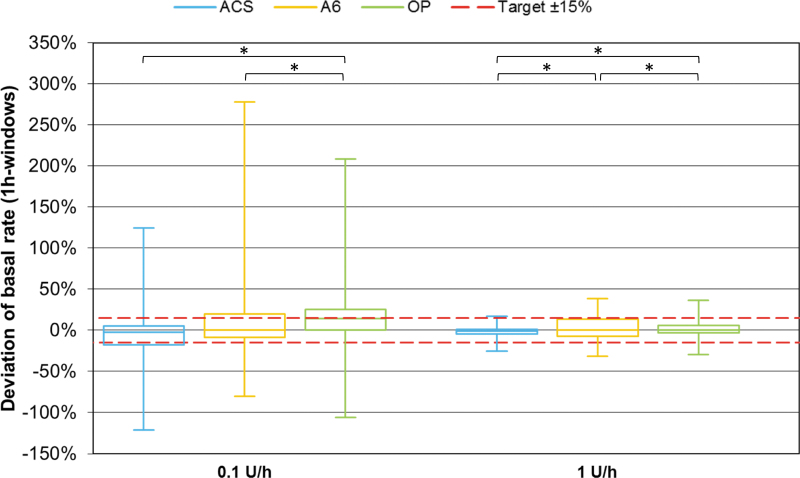
One-hour window basal rate delivery accuracy of different pumps grouped by basal rate (*n* = 648 from nine repetitions per pump). Red dashed lines represent target delivery ±15%. Asterisks indicate statistically significant differences in percentage of 1-h windows within ±15% of the target between pumps (*χ*^2^-tests; *P* < 0.025).

**FIG. 6.**
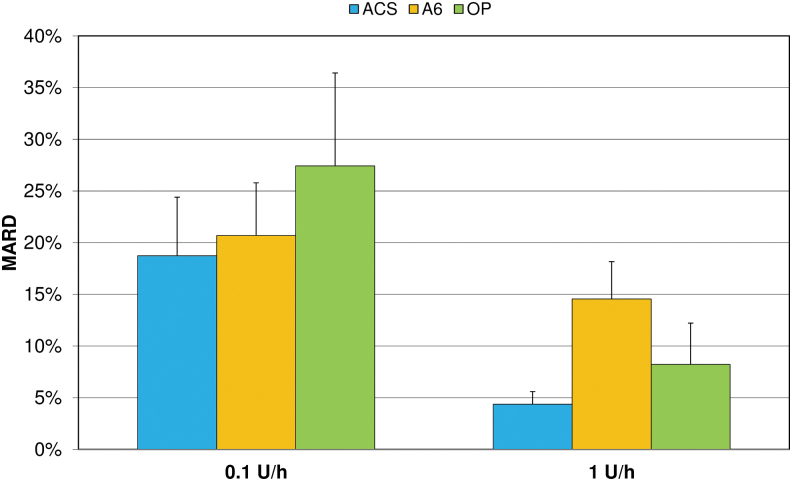
Mean absolute relative differences (MARD) of 1-h windows from target delivery during 72 h basal rate delivery (mean and SD of nine repetitions).

Considering basal rate accuracy, ACS showed significantly more 1-h windows within ±15% of the target than OP at both tested basal rates (*P* < 0.001) and more than A6 only at the larger basal rate (*P* < 0.001) (Fig. 5). In addition, OP showed fewer and less pronounced deviations from target than A6 at 1 U/h, but more and larger deviations at 0.1 U/h (*P* < 0.001). Similarly, MARD was lowest for ACS (18.7% at 0.1 U/h and 4.4% at 1 U/h) compared with A6 (20.7% and 14.5%) and OP (27.4% and 8.2%) (Fig. 6).

**FIG. 7.**
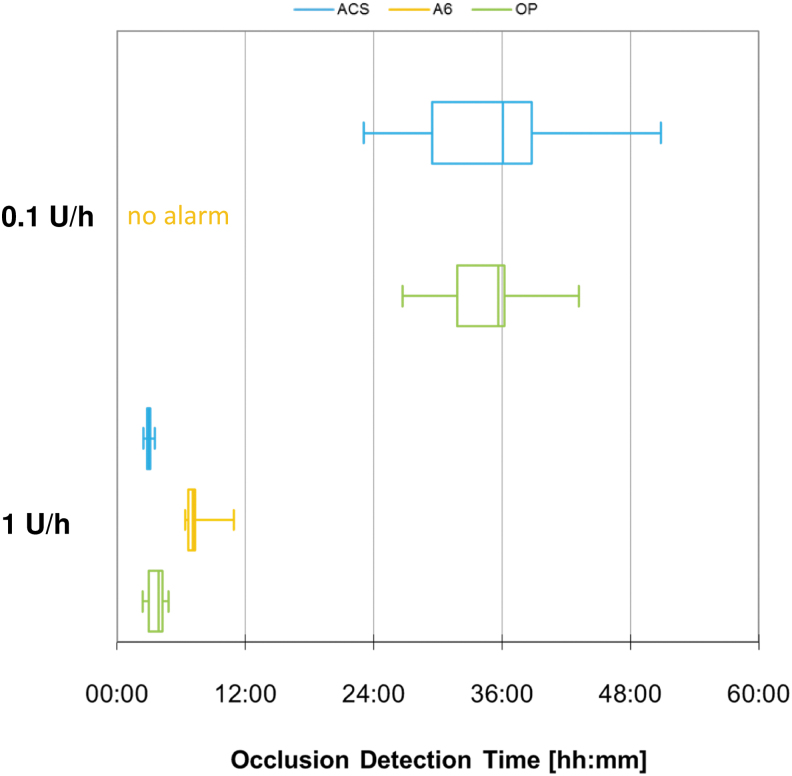
Occlusion detection time of different pumps grouped by basal rate (*n* = 9 repetitions).

Occlusion detection was comparable between ACS and OP and slower or not present for A6 (Fig. 7). However, when the 0.1 U/h basal rate was run, mean occlusion detection time was >24 h for all pumps.

## Discussion

In this evaluation, different currently available tubeless pumps were tested and compared. These are the latest results from a series of tests that are constantly updated when new insulin pumps become available. The two new pumps were recently introduced and represent alternatives to OP for patients who prefer a tubeless insulin pump with advanced features. To our knowledge, no independent tests of ACS and A6 have been published previously. Most tubeless pump accuracy tests are limited to OP, as this pump is widespread. Other tubeless pumps that were previously tested in other evaluations such as the JewelPump™ ^6^ (Debiotech) or the Cellnovo insulin infusion system^8^ (Cellnovo, withdrawn from the market in 2019) were not available for this test series.

An adequate test setting for tubeless pumps is still under debate, especially as conflicting results are reported for OP from different working groups using different experimental designs.^4–11^ In our previous tests, OP was only compared with durable pumps and showed inferior accuracy, giving rise to doubts about the experimental set up. However, since the same delivery patterns (large and recurrent variation between 1-h windows) were only observed in one of the two new pumps tested with the same setup, and as parts of the newly obtained results were comparable with those of durable pumps, these methodological doubts are now diminished.

In the present analysis, increasing accuracy with larger insulin doses was observed for the new pumps for bolus as well as basal rate delivery, confirming several other investigations.^9–11^

The mean deviation of boluses from target was <5% for all bolus sizes in all pumps. Manufacturers of A6 and OP claim an accuracy of ±5% for all bolus sizes, whereas ACS specifies ±5% only for 50 U boluses and ±30% for 0.2 U. This means that the mean deviation of boluses was within specification for all pumps. However, individual boluses showed larger deviations with up to 60% of boluses deviating >15% and it is this dose-to-dose variability that may impact on glucose variation and type 1 diabetes self-management efficacy. The ACS manufacturer specified a much larger acceptable range for the smallest bolus; all of the assessed boluses fell within their specifications. Irrespective of predefined specifications, ACS showed significantly more boluses within ±15% of the target than the other pumps.

When the mean values are calculated, positive and negative deviations might be balanced and only systematic errors become obvious. The MARD includes absolute deviations and might, therefore, provide a better estimation of the bolus function in practice. Maximal MARDs for boluses of different sizes among the three pumps ranged from <5% to 20% and highlighted the differences between pumps. Clinical impact, however, may be more apparent from random errors and scatter. Therefore SD, which emphasizes the variance of boluses, should always be considered. An insulin pump showing a smaller SD might be preferred to a pump only showing a small mean difference from the expected insulin delivery.

The same principles apply to basal rate delivery. Overall basal rate delivery was within ±12% of the intended delivery after 72 h for all pumps; however, hourly intervals deviated considerably more. The rate of 1-h windows within ±15% of the target was similar for the new pumps at the 0.1 U/h basal rate, but higher than that previously reported for OP.^10^ At 1 U/h, however, ACS showed a significantly more precise delivery. Large positive and negative variations between single deliveries or short intervals have been previously reported with OP and were also observed for A6 at the larger basal rate.^7^

Although none of the tubeless pumps tested fully matched the accuracy of the best durable pumps for all parameters tested,^10^ the results of our evaluation indicate that acceptable accuracy can be achieved by tubeless pumps, with different pumping or manufacturing mechanisms potentially determining delivery performance. As Girardot et al. recently reported, lowering of basal rates is handled differently by different pumps.^11^ Although some pumps reduce the amount of insulin delivered with each burst, others reduce the frequency of bursts.

Whether, and in what way, the inaccuracies observed in these in vitro tests affect clinical outcomes remains to be clarified, for example, by simulation models. However, it is expected that fluctuations in delivery have a higher impact than systematic over- or underdeliveries as these are easier to adjust for. Even though pharmacokinetics of subcutaneously applied insulin might buffer transient inaccuracies, unsatisfactory glucose control despite CSII might reflect pump performance, accuracy, and precision compounding errors in glucose measurement and carbohydrate estimations. With regard to the use of pumps in artificial pancreas systems, short-term accuracy is especially important, because insulin delivery is frequently adapted to current glucose levels.^18^ In addition, available hybrid closed-loop systems deliver so-called microboluses instead of a basal rate, that is, very small boluses are delivered every few minutes.^19^ However, in a recent artificial pancreas study, satisfactory glycemic control could be shown with OP,^20^ suggesting that feedback control may be able to compensate for inaccuracy and variance in insulin delivery.

Tubeless pumps are popular among children, because without an external infusion set, there is less risk of kinking or unintended pulling out.^2^ The new tubeless pumps presented here are intended to be used by children who are at least 2 years old and OP does not specify age restrictions. Because of the low insulin requirement, very small basal rates are used in small children and especially in infants. The larger inaccuracies observed at smaller insulin doses and related risks should thus always be considered during CSII in children.

Occlusion detection is a safety mechanism of insulin pumps and alarmingly is not as reliable as one would expect. It is already known that occlusion alarms occur after an extended period with nearly all pumps, especially when small basal rates are used.^17^ However, for A6 no alarm was registered at 0.1 U/h at all. In addition, the occlusion detection time at 1 U/h was higher than in other pumps. This strengthens the recommendations for regular self-monitoring of glucose to detect elevated glucose levels that might indicate an occlusion, and support and education, including sick day rules.^21^

## Conclusion

The results of our evaluation indicate that insulin delivery accuracy significantly differs between the tubeless pump models tested. The smaller the volumes to be delivered, the larger are the observed deviations, especially over shorter time periods. In addition, occlusion alarms are an issue when very small basal rates are used. However, the most accurate tubeless pump tested was almost as accurate as previously tested durable pumps.

## Supplementary Material

Supplemental data

Supplemental data
