# Supplementary material for: Evaluation of the Accuracy of Current Tubeless Pumps for Continuous Subcutaneous Insulin Infusion
Source: Diabetes Technol Ther. 2021 Apr 20;23(5):350–7. doi: 10.1089/dia.2020.0525 (PMC8080918; doi:10.1089/dia.2020.0525)
Supplement: Supplemental data [file Supp_Table1.docx]

Supplementary Table 1: Device-to-device variation: Accuracy of bolus delivery at different bolus volumes for each measurement (n=25 for 0.2 U and 1 U; n=12 for 10 U)

| **Insulin pump** | **Measurement** | **0.2 U** | | **1 U** | | **10 U** | |
| --- | --- | --- | --- | --- | --- | --- | --- |
|  |  | **Mean deviation ± SD** | **Individual boluses within ± 15%** **of the target** | **Mean deviation ± SD** | **Individual boluses within ± 15%** **of the target** | **Mean deviation ± SD** | **Individual boluses within ± 15%** **of the target** |
| ACS | 1 | 0.1% ± 5.6% | 96% | 0.2% ± 8.7% | 100% | 0.1% ± 1.0% | 100% |
|  | 2 | -2.3% ± 11.4% | 84% | 0.9% ± 8.4% | 92% | 0.0% ± 0.9% | 100% |
|  | 3 | -3.1% ± 7.0% | 96% | 0.0% ± 6.4% | 96% | -0.3% ± 0.7% | 100% |
|  | 4 | -3.2% ± 7.1% | 92% | -0.3% ± 3.1% | 100% | -0.1% ± 0.8% | 100% |
|  | 5 | -3.9% ± 6.6% | 92% | 0.6% ± 2.5% | 100% | -0.3% ± 0.9% | 100% |
|  | 6 | -5.9% ± 9.7% | 80% | 0.3% ± 5.5% | 100% | 0.0% ± 0.7% | 100% |
|  | 7 | -4.3% ± 9.9% | 76% | 0.2% ± 5.6% | 100% | 0.4% ± 0.9% | 100% |
|  | 8 | -0.9% ± 7.9% | 92% | 0.6% ± 2.7% | 100% | 0.0% ± 0.8% | 100% |
|  | 9 | -5.9% ± 8.0% | 88% | 0.6% ± 2.1% | 100% | 0.3% ± 0.6% | 100% |
| A6 | 1 | 4.0% ± 24.4% | 44% | 4.8% ± 16.1% | 56% | 3.2% ± 0.6% | 100% |
|  | 2 | 1.1% ± 19.6% | 36% | 4.8% ± 18.8% | 76% | 3.5% ± 0.9% | 100% |
|  | 3 | 3.1% ± 21.7% | 32% | 3.6% ± 17.7% | 60% | 2.8% ± 0.6% | 100% |
|  | 4 | 2.1% ± 26.0% | 32% | 4.0% ± 10.3% | 72% | 2.9% ± 0.7% | 100% |
|  | 5 | 3.7% ± 23.0% | 52% | 3.8% ± 19.5% | 60% | 3.3% ± 0.8% | 100% |
|  | 6 | 2.3% ± 29.0% | 20% | 4.1% ± 15.9% | 60% | 2.6% ± 1.0% | 100% |
|  | 7 | 3.0% ± 18.7% | 52% | 4.3% ± 14.1% | 68% | 3.2% ± 0.7% | 100% |
|  | 8 | 2.2% ± 29.2% | 32% | 3.5% ± 14.7% | 80% | 3.1% ± 0.9% | 100% |
|  | 9 | 7.7% ± 18.8% | 56% | 3.1% ± 17.1% | 52% | 3.0% ± 0.7% | 100% |
| OP | 1 | 3.0% ± 11.4% | 72% | 0.0% ± 21.4% | 60% | 0.2% ± 0.6% | 100% |
|  | 2 | 1.9% ± 10.0% | 88% | 0.4% ± 4.3% | 100% | -0.2% ± 0.5% | 100% |
|  | 3 | 1.2% ± 20.1% | 32% | 0.9% ± 3.5% | 100% | 0.2% ± 0.4% | 100% |
|  | 4 | -0.2% ± 33.1% | 44% | -2.8% ± 18.5% | 32% | 0.4% ± 0.9% | 100% |
|  | 5 | 3.4% ± 21.9% | 44% | 0.3% ± 11.5% | 84% | -0.1% ± 0.7% | 100% |
|  | 6 | 1.9% ± 15.6% | 68% | 1.0% ± 5.1% | 100% | 0.2% ± 1.1% | 100% |
|  | 7 | -0.7% ± 17.7% | 64% | -1.0% ± 7.8% | 92% | 0.5% ± 0.5% | 100% |
|  | 8 | 0.9% ± 27.3% | 48% | 0.2% ± 15.3% | 60% | 0.4% ± 0.6% | 100% |
|  | 9 | 2.3% ± 23.0% | 56% | 1.0% ± 13.2% | 64% | 0.6% ± 0.6% | 100% |
