# Supplementary material for: Evaluation of the Accuracy of Current Tubeless Pumps for Continuous Subcutaneous Insulin Infusion
Source: Diabetes Technol Ther. 2021 Apr 20;23(5):350–7. doi: 10.1089/dia.2020.0525 (PMC8080918; doi:10.1089/dia.2020.0525)
Supplement: Supplemental data [file Supp_Table2.docx]

Supplementary Table 2: Device-to-device variation: Accuracy of basal rate delivery at different basal rates for each measurement (72 h divided into 1-h windows)

| **Insulin pump** | **Measurement** | **0.1 U/h** | | | **1 U/h** | | |
| --- | --- | --- | --- | --- | --- | --- | --- |
|  |  | **Mean total deviation** | **SD (1-h windows)** | **1-h windows within ± 15% of the target** | **Mean total deviation** | **SD (1-h windows)** | **1-h windows within ± 15% of the target** |
| ACS | 1 | 3.7% | 20.3% | 75% | -1.8% | 5.6% | 97% |
|  | 2 | 7.1% | 20.6% | 76% | -1.3% | 4.5% | 100% |
|  | 3 | 0.6% | 12.8% | 75% | -2.3% | 8.7% | 90% |
|  | 4 | -17.9% | 25.3% | 27% | -1.5% | 3.9% | 100% |
|  | 5 | -10.3% | 30.8% | 45% | -2.2% | 4.4% | 100% |
|  | 6 | -13.5% | 21.6% | 38% | -3.2% | 6.5% | 97% |
|  | 7 | -3.3% | 22.2% | 39% | -1.8% | 4.1% | 100% |
|  | 8 | -2.3% | 32.6% | 41% | -1.6% | 5.6% | 99% |
|  | 9 | -12.4% | 21.9% | 39% | -1.8% | 4.1% | 99% |
| A6 | 1 | 2.5% | 30.2% | 46% | 3.1% | 18.5% | 60% |
|  | 2 | 4.4% | 47.3% | 48% | 3.0% | 20.3% | 37% |
|  | 3 | 0.3% | 21.8% | 54% | 1.1% | 9.3% | 87% |
|  | 4 | 8.6% | 29.9% | 49% | 4.1% | 22.2% | 51% |
|  | 5 | 5.7% | 19.7% | 61% | 4.9% | 12.9% | 71% |
|  | 6 | 8.1% | 17.5% | 61% | 3.3% | 19.0% | 39% |
|  | 7 | 4.3% | 20.0% | 54% | 4.6% | 17.2% | 77% |
|  | 8 | 10.9% | 23.9% | 35% | 3.5% | 15.5% | 50% |
|  | 9 | -3.2% | 31.4% | 28% | 2.6%^1^ | 13.3%^1^ | 66%^1^ |
| OP | 1 | 15.9% | 20.9% | 41% | 1.1% | 5.6% | 99% |
|  | 2 | -12.7% | 60.2% | 17% | 0.0% | 6.9% | 97% |
|  | 3 | 16.0% | 24.6% | 28% | 0.9% | 9.9% | 87% |
|  | 4 | 18.0% | 30.4% | 34% | 1.4% | 13.1% | 65% |
|  | 5 | 20.8% | 33.8% | 27% | 1.4% | 17.2% | 54% |
|  | 6 | -6.7% | 47.1% | 34% | 1.0% | 5.1% | 99% |
|  | 7 | 20.2%^1^ | 17.3%^1^ | 26%^1^ | 1.5% | 15.2% | 63% |
|  | 8 | 16.7% | 22.5% | 42% | 2.4% | 13.0% | 70% |
|  | 9 | 18.4% | 26.2% | 35% | 1.9% | 6.4% | 97% |

^1^ measurement was stopped earlier due to an error notice
